# A Role for Myocilin in Receptor-Mediated Endocytosis

**DOI:** 10.1371/journal.pone.0082301

**Published:** 2013-12-18

**Authors:** Brian S. McKay, Nicole R. Congrove, Adiv A. Johnson, W. Michael Dismuke, Trent J. Bowen, W. Daniel Stamer

**Affiliations:** 1 Department of Ophthalmology and Vision Science, University of Arizona, Tucson, Arrizona; 2 Department of Cellular and Molecular Medicine, University of Arizona, Tucson, Arrizona; 3 Department of Ophthalmology, Duke University, Durham, North Carolina; 4 Department of Biomedical Engineering, Duke University, Durham, North Carolina; Casey Eye Institute, United States of America

## Abstract

Myocilin is a broadly expressed protein that when mutated uniquely causes glaucoma. While no function has been ascribed to explain focal disease, some properties of myocilin are known. Myocilin is a cytoplasmic protein that also localizes to vesicles specifically as part of a large membrane-associated complex with properties similar to the SNARE machinery that function in vesicle fusion. Its role in vesicle dynamics has not been detailed, however myocilin intersects with the endocytic compartment at the level of the multivesicular body. Since internalized GPCRs are sorted in the multivesicular body, we investigated whether myocilin functions in ligand-dependent GPR143 endocytosis. Using recombinant systems we found that the kinetics of myocilin recruitment to biotinylated membrane proteins was similar to that of arrestin-3. We also co-localized myocilin with GPR143 and Arrestin-2 by confocal microscopy. However, wild-type myocilin differed significantly in its association kinetics and co-localization with internalized proteins from mutant myocilin (P370L or T377M). Moreover, we found that myocilin bound to the cytoplasmic tail of GPR143, an interaction mediated by its amino terminal helix-turn-helix domain. Hydrodynamic analyses show that the myocilin-GPR143 protein complex is >158 kD and stable in 500 mM KCl, but not 0.1% SDS. Collectively, data indicate that myocilin is recruited to the membrane compartment, interacting with GPCR proteins during ligand-mediated endocytosis and that GPCR signaling underlies pathology in myocilin glaucoma.

## Introduction

Myocilin is 504 amino acid protein expressed by different tissues and cell-types in the body [1]. For example, muscle cells, neurons, fibroblasts, endothelia and various epithelia all express myocilin [2]–[5]. Despite a widespread expression profile, myocilin’s function remains elusive and mutations in myocilin result in a very limited, but clinically important phenotype of ocular hypertension and glaucoma [6]–[10]. Only retinal ganglion cells and tissues that control intraocular pressure appear adversely affected in a dominant negative manner by mutations in myocilin. Interestingly, myocilin ablation in mice results in no detectible ocular or systemic phenotype [11], suggesting that myocilin is not essential and that there is functional redundancy by other proteins.

In all cell types examined, myocilin localizes to a vesicular compartment [12], [13]. Some studies suggest that myocilin is vesicular cargo as an extracellular matrix protein [14]–[17]. However, about one half of myocilin is cytosolic (not membrane or vesicle associated) [18] and only a subpopulation of cells release myocilin into the extracellular space [1], [4], [19], [20] suggesting it is not cargo within the vesicles. An alternative, supported by other studies, point to a role for myocilin in vesicular trafficking [12]. For example, myocilin does not contain a functional signal peptide [2], [12], [21] or a transmembrane domain [2], two known requirements for a protein to be either carried as vesicular cargo or localize as an integral membrane protein, respectively. Rather, myocilin appears to be a peripheral membrane protein, part of a >405 kD membrane-associated complex, sharing characteristics with the SNARE machinery that functions in vesicle fusion [18]. Namely, the myocilin complex is SDS-resistant, centered about coiled-coil interactions, and similar in retinal neurons and the retinal pigment epithelium, indicating a lack of tissue specificity.

Members of the SNARE machinery are critical to the formation and fusion of membrane vesicles [22], [23]. The SNARE complex is a large 20s complex of membrane associated proteins that contains both cytoplasmic and transmembrane proteins, and the protein:protein interactions in this complex are resistant to disruption by SDS [24]. Different isoforms of members of the SNARE complex govern the specificity of and timing of vesicle fusion events [25]. Fusion occurs when a SNARE protein on the target membrane binds to a complimentary SNARE protein on the vesicle, and the folding of the SNAREs together drives membrane fusion.

Myocilin appears to function somewhere in the endocytic pathway. Unlike constitutively released extracellular cargo, myocilin is released within minutes upon stimulation by cells on the surface of exosomes [19], [26], [27]. Exosomes are nanovesicles and a component of the signal transduction machinery that traffics through the multivesicular body (MVB) [28]–[32]. While the precise role of the MVB is unclear, the MVB appears to function as a sorting organelle for vesicles in the endocytic pathway [33]. Endocytic vesicles arise primarily from internalization of the plasma membrane and can down regulate signaling activity of ligand-bound plasma membrane receptors. Depending upon context, endocytic vesicles are recycled back to the plasma membrane, targeted to the late endosomes/MVB or sent to the lysosome compartment for degradation [30], [34]. Exosomes derived from the MVB membranes have myocilin on their surface [13], [19], suggesting the possibility that myocilin enters the vesicular pathway at the point of receptor endocytosis. Arrestin proteins function in the process of receptor-mediated endocytosis [35], [36], where they are recruited to the membrane after ligand stimulation and begin to form the scaffold of proteins that coalesce the activated receptors. The aggregated receptors develop a scaffold of proteins that deform the membrane, then pinch of the membrane with the receptors into an endosome [37], [38]. Endosomes are trafficked through the cytoplasm to multiple fates, and arrestin is released from early endosomes during receptor sorting steps.

In the present study, we tested the hypothesis that persistent G-protein-coupled receptor (GPCR) activation results in receptor internalization and myocilin recruitment to the endocytic pathway, similar to arrestin. Our results indicate that, independent of cell-type or species, myocilin is recruited to the vesicular pathway during ligand-stimulated endocytosis of the G-protein-coupled receptor, GPR143. Myocilin recruitment is transient, peaking at approximately 20 minutes after ligand stimulation. Binding of myocilin to GPR143 occurs between the cytoplasmic carboxyl tail of GPR143 and the amino terminus of myocilin as part of a protein complex (>158 kD). Together our data indicate that myocilin is a protein that functions in the endocytic pathway during receptor endocytosis.

## Experimental Procedures

### Cell Culture

MCF7, COS-7, and CHO cells (obtained from ATCC) were cultured in Dulbecco's Modified Eagle Medium (DMEM) supplemented with 5% FBS, 1% antibiotic/antimycotic, and maintained at 37°C in humidified air containing 5% CO_2_. Low tyrosine (LT) medium was used in experiments examining GPR143 signaling [39]. LT medium contains tyrosine-free DMEM supplemented with 5 µM tyrosine and 5% dialyzed FBS. All cell culture reagents were obtained from Invitrogen Life Technologies.

### Biotinylation and Capture of Cell Surface Proteins

Cells were rinsed three times with ice cold reaction buffer (100 mM NaCl, 50 mM NaHCO_3_, pH 7.45) then incubated for two 30 min reactions using either Sulfo-NHS-LC Biotin or Sulfo-NHS-SS Biotin (1.0 mg/ml, Thermo Scientific). Following the biotinylation, cells were returned to culture medium at 37°C for 10 minutes prior to stimulation with1 µ M 3, 4-dihydroxyphenylalanie (l-DOPA) (Sigma). After stimulation, residual biotin on cell surfaces was cleaved using 100 mM 2-mercaptoethanesulfonate in DMEM (2×15 min at 4°C) for experiments using Sulfo-NHS-SS Biotin. To capture the remaining biotinylated proteins cells were harvested in lysis buffer (2 mM EDTA, 1% Triton X100, and 1% Tween 20 in Tris Based Saline Buffer, pH 7.4) containing Halt Protease Inhibitor Cocktail (Thermo Scientific). Intact cells and insoluble debris were removed by centrifugation at 14,000 rpm for 20 min. Biotinylated proteins were captured overnight with immobilized streptavidin cross-linked agarose resin (Thermo Scientific) and then mixed with 4x reducing buffer (250 mMTris, pH 6.8, 8% SDS, 40% glycerol, 20% beta-mercaptoethanol, 0.08% bromophenol blue).

### Transfections

The construction and characterization of plasmid DNAs encoding full-length myocilin (WT, P370L, T377M) and individual myocilin domains have been described previously [12]. Plasmid DNAs encoding GPR143 and its cytoplasmic domain have been described previously [39]. For experiments in which myocilin GFP fluorescence was observed, we used GPR143 expressed in the pSport6 expression vector (Invitrogen Life Technologies). The arrestin-2 GFP plasmid was a kind gift or Dr. Robert Lefkowitz, Duke University. FuGENE 6 reagent was used for transfection of plasmid DNA into cells per manufacturer instruction (Promega). Geneticin (250 µ µg/ml, Invitrogen) was used for selection and establishing stable expression of recombinant protein in transfected cells.

### Western blotting and Silver Staining

Solubilized proteins or cell conditioned medium (CM), collected after 48 hours with the cells, were separated by SDS-PAGE then transferred electrophoretically to nitrocellulose membranes. Non-specific sites on membranes were blocked with 5% nonfat dry milk in Tris-buffered saline, 0.2% Tween 20 (TBST). Rabbit anti-myocilin antibodies or monoclonal antibodies that specifically recognize β-actin (Abcam) were incubated with membranes overnight at 4°C. Polyclonal antibodies directed against arrestin-3 (β-arrestin-2), produced in goat were purchased from Abcam. Polyclonal rabbit anti-myocilin antibodies were produced, affinity purified and characterized by our laboratory [19], [40]. Membranes were washed in TBST, 3 times for 20 minutes, incubated with anti-rabbit or anti-mouse horseradish peroxidase-conjugated secondary antibodies (Jackson ImmunoResearch Labortories, Inc.,West Grove, PA) in TBST for 1 hour, and washed with TBST 3 times for 10 minutes. Enhanced chemiluminescence (ECL) (Amersham Biosciences) and X-ray film (Phenix Research Products, Belgium) were used to visualize protein-antibody complexes. For silver staining, protein standards (GE healthcare, Buckinghamshire, UK) were separated by SDS-PAGE and stained using a Bio-Rad silver stain kit per the manufactures instructions. Band densitometry was obtained using ImageJ and comparative analysis was accomplished using paired t-tests where p<0.05 was considered statistically significant.

### Gradient Sedimentation

Equal protein fractions in phosphate buffered saline (150 mM NaCl, 25 mM NaHCO_3_, 2 mM CaCl_2_) or PBS adjusted to 0.1% SDS, or adjusted to 500 mM KCl were layered onto linear glycerol gradients (10–30%) and centrifuged at 100,000 g, 25°C for 2 hours in a TLS-55 rotor (Beckman Coulter, USA). Fractions were collected from the top and western blotting was used to detect myocilin content in the collected fractions as described previously [18]. Svedberg markers run in a separate gradient were ribonuclease A, aldolase, conalbumin, and ovalbumin proteins from GE Healthcare.

### Confocal Microscopy

Transfected stable MCF7 and CHO cells were grown on a sterile glass slides and biotinylated as described above. The cells were washed with 1× TBST, and fixed overnight at 4°C with 4% (w/v) paraformaldehyde. After three additional washing steps with 1× TBST, cells were stained with antibodies to myocilin followed by TRITC conjugated secondary to rabbit (Jackson ImmunoResearch Labortories, Inc.,West Grove, PA ). To visualize conjugated proteins, fixed cells were stained with 1:2000 conjugated rhodamine streptavidin (Thermo Scientific). The cells were washed with 1x TBST and mounted. Pictures were taken with a 40X oil immersion objective using a Leica SP5 confocal microscope. GFP fluorescence was excited using 488nm laser illumination, with emission detected between 496–552nm, TRITC fluorescence was excited using 543nm laser illumination and emission was detected between 557–621nm. Photomicrographs were produced using full cell thickness scans, 308nm depth per section, then digital flattening the image stack.

### GPR143 Purification

Affinity chromatography was utilized for purification of GPR143:maltose binding protein (MBP) and MBP using amylose resin (NEB), performed according to the manufacturer’s directions. To capture myocilin, or the N-terminal domain (HtH, 58 amino acids) of myocillin, COS cells expressing the proteins were harvested in 1% NP40, 5% CHAPS in PBS with Halt Protease Inhibitor Cocktail (Thermo Scientific). Intact cells and fragments were removed by centrifugation at 25,000 x g for 25 minutes at 4°C. Homogenates were batch adsorbed overnight to GPR143:MBP bound amylose resin or amylose resin with bound MBP alone. The column was washed 12x with 1% NP40, 5% CHAPS in PBS and bound proteins were eluted by competition with 10 mM maltose.

## Results

### Myocilin co-localizes with GPR143 and arrestin-2 in ligand dependent manner

**Figure 1:**
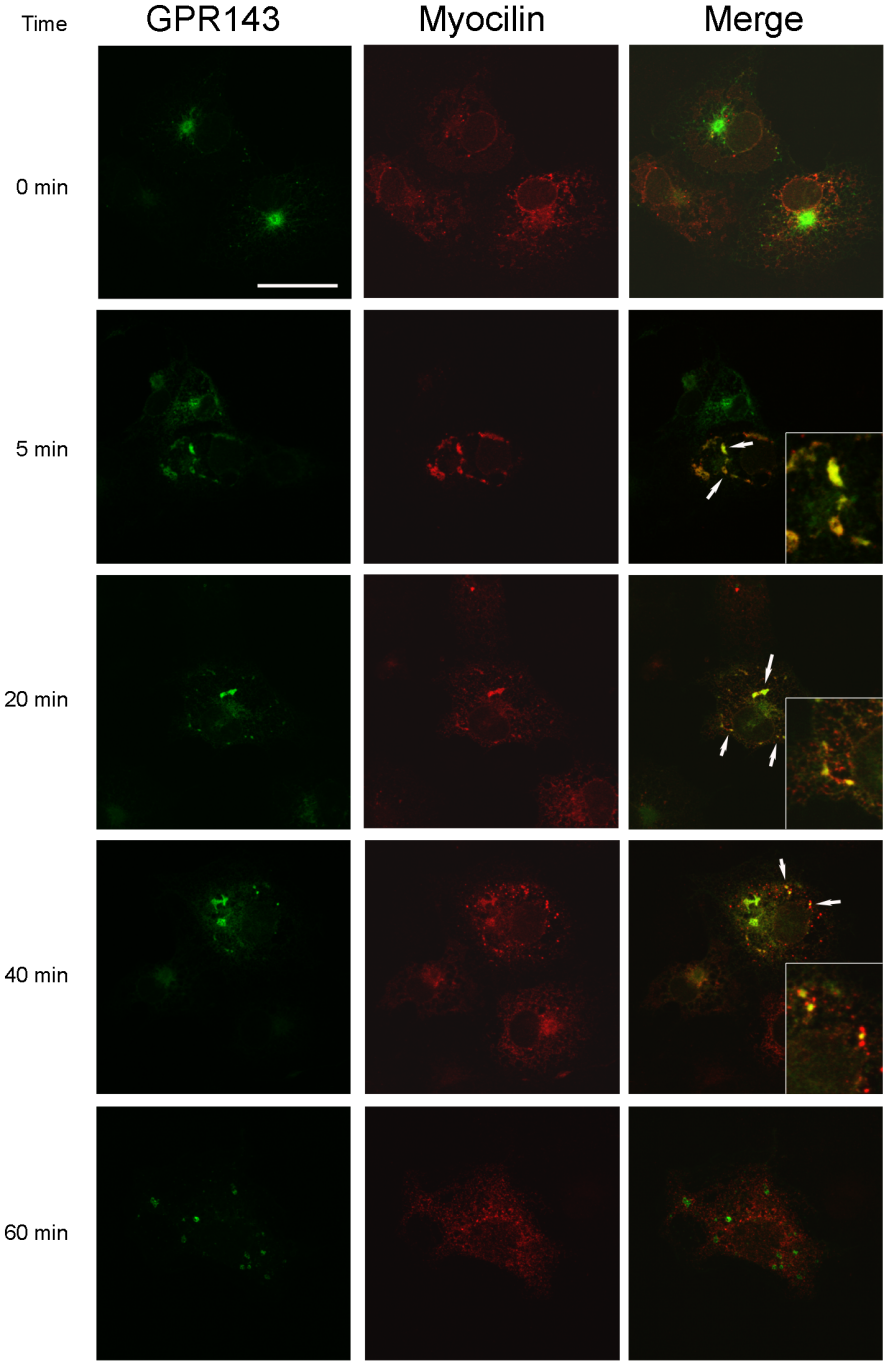
Ligand-dependent co-localization of myocilin and GPR143. Confocal micrographs of GPR143 linked to GFP and myocilin identified by immunofluorescence. Cells were harvested at 0, 5, 20, 40, 60 after exposure to 1 µM l-DOPA. Overlaid images illustrate co-localization of GPR143 and myocilin starting at 5 minutes and continuing through 20 minutes, with little co-localization at 40 minutes and no co-localization at time 0 and 60 minutes. Insets (shown for 5, 20 and 40 min) are 4x computer magnification of a region denoted with an arrow illustrating co-localization. Bar equals 25 µM.

COS cells were transfected to express GPR143-GFP and unconjugated myocilin, then stimulated with its endogenous ligand, L-DOPA, to promote receptor-mediated endocytosis (Figure 1). Cells were stained using an antibody to myocilin and a TRITC secondary to visualize myocilin (red). At the starting point, time 0, there is not discernable co-localization between myocilin and GPR143. However, as soon as 5 minutes after L-DOPA stimulation, there is clear co-localization between the two proteins. The interaction between myocilin and GPR143 continues as receptors are internalized, notice the 20 minutes time point, but the two proteins begin to traffic separately by 40 minutes. At 60 minutes myocilin distribution is vesicular and similar to time 0, basal conditions, whereas GPR143 is present in a large cytoplasmic endosome.

**Figure 2:**
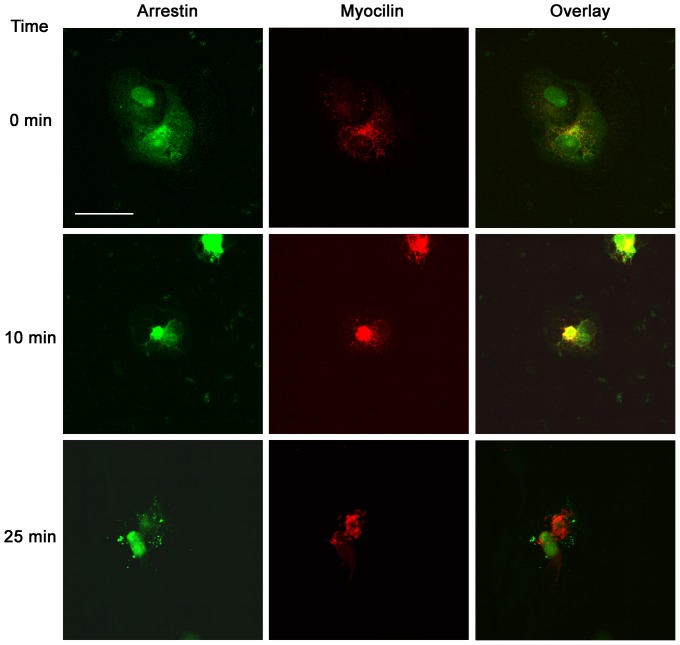
Co-localization of myocilin and arrestin 2. Confocal micrograph of arrestin 2 linked to GFP and myocilin observed by immunofluorescence at time 0, 10, and 25 minutes after l-DOPA treatment. The images are overlaid to illustrate co-localization at 10 and lack of co-localization at 0 and 25 minutes. Bar equalsµM.

Myocilin also co-localizes with arrestin 2. In this experiment we transfected COS cells to express arrestin-2 conjugated to GFP as well as unconjugated GPR143 and myocilin. As above, myocilin was visualized after immunostaining (red) while arrestin-2 GFP is presented in green (Figure 2). After 10 minutes of continuous l-DOPA stimulation we observed arrestin-2 and myocilin co-localize at the apical membrane in a patch. With both arrestin-2 and GPR143 at the 10 minute time point we observed cells with small vesicles near the membrane (as shown for GPR143) and cells with an apical patch (as shown for arrestin-2). However, results differed at the 25-minute time point. Arrestin-2 and myocilin illustrate very little co-localization after 25 minutes of stimulation, just a few small vesicles near the large central endosomal compartment. At this time arrestin-2 is largely absent from the central endosomal compartment where myocilin has accumulated.

### Co-localization of myocilin with endocytosed membrane proteins

**Figure 3:**
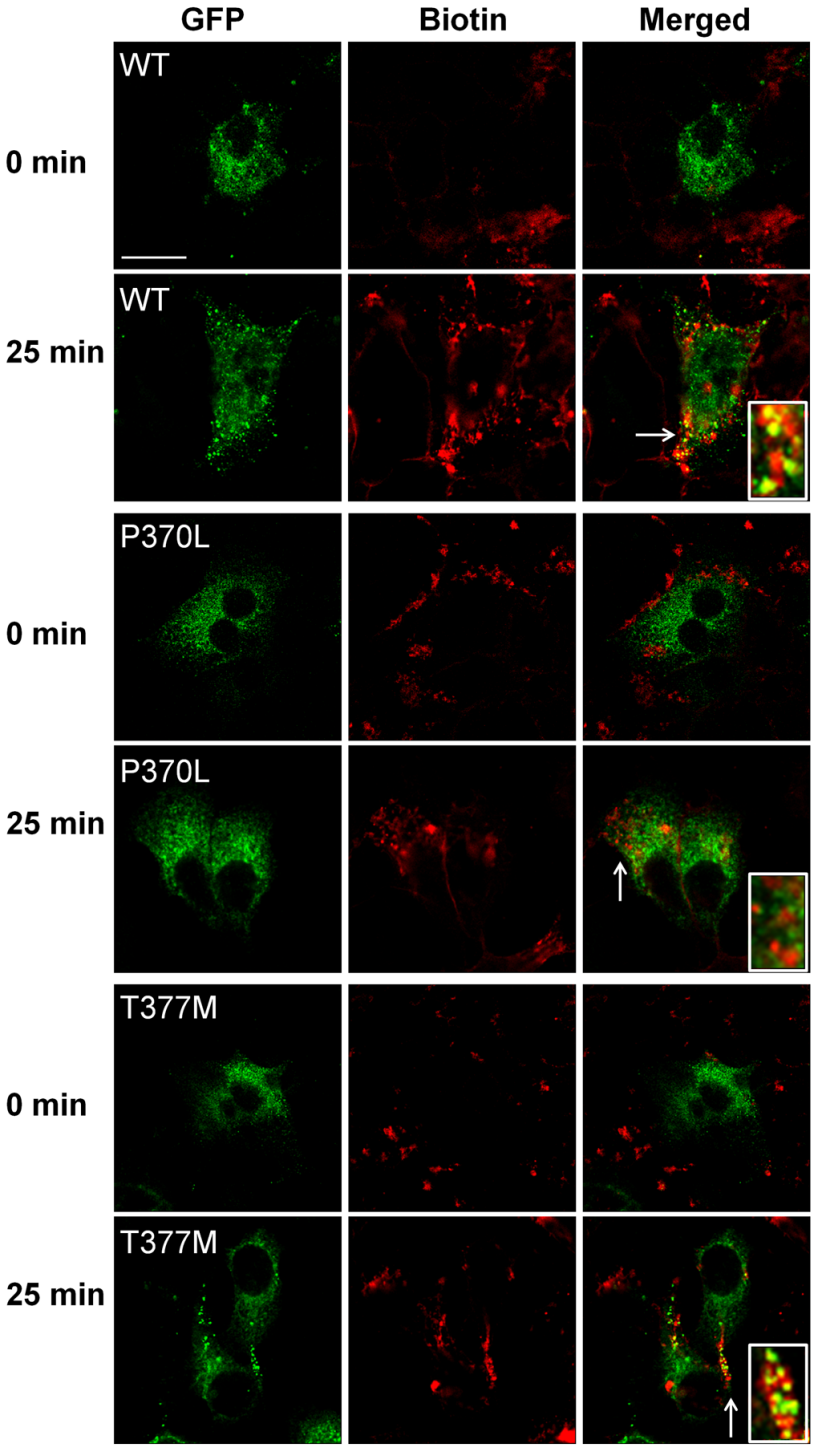
Co-localization of myocilin and endocytosed membrane proteins after GPCR stimulation. Cell surface proteins of MCF7 cells expressing myocilin (WT, P370L, or T377M) linked to GFP (green) were biotinylated. Cells were stimulated for 25 minutes with L-DOPA or remained untreated (time zero), and residual cell surface biotin was cleaved by disulfide reduction. Cells were fixed, labeled with rhodamine streptavidin (red) and imaged by confocal microscopy. Insets represent a 4-fold magnification of the cellular region identified by the arrow. Results are representative of 5 experiments conducted in MCF7 cells. Bar  =  25 µM.

Using confocal microscopy, myocilin participation in ligand-stimulated endocytosis was monitored over time using a second method. MCF7 cells stably transfected with GPR143 plus GFP-tagged myocilin (WT, P370L or T377M) were placed on ice to facilitate labeling of cell surface proteins and inhibit internalization, then biotinylated on their cell surface. Cells were returned to warm culture media for 10 min and then treated with l-DOPA. Cells were fixed at 25 minutes after treatment and compared to untreated controls. Just prior to fixation, the residual cell surface biotin was removed by reduction of the disulfide bond contained in the biotin label, leaving predominantly internalized biotin-labeled proteins with little cell surface biotin remaining. Localization of biotin-labeled proteins was compared to GFP- myocilin. Significant co-localization between any of the myocilin isoforms and internalized biotinylated proteins was not observed in untreated, time zero controls. However, twenty-five minutes after stimulation with l-DOPA, WT myocilin co-localized with a subpopulation of endocytic vesicles containing biotinylated cell surface proteins (Figure 3). The general distribution of myocilin-associated vesicles changed from perinuclear to a more peripheral distribution at the cell borders. Co-localization with endocytic vesicles was greatest at the edge of the cell borders. The appearance of the T377M myocilin was similar to WT myocilin 25 minutes after treatment, becoming more peripheral, with some co-localization. In contrast, the localization of the P370L isoform did not change after treatment and did not co-localize with biotinylated proteins in endosomes.

### Kinetics of myocilin association with biotinylated membrane proteins

**Figure 4:**
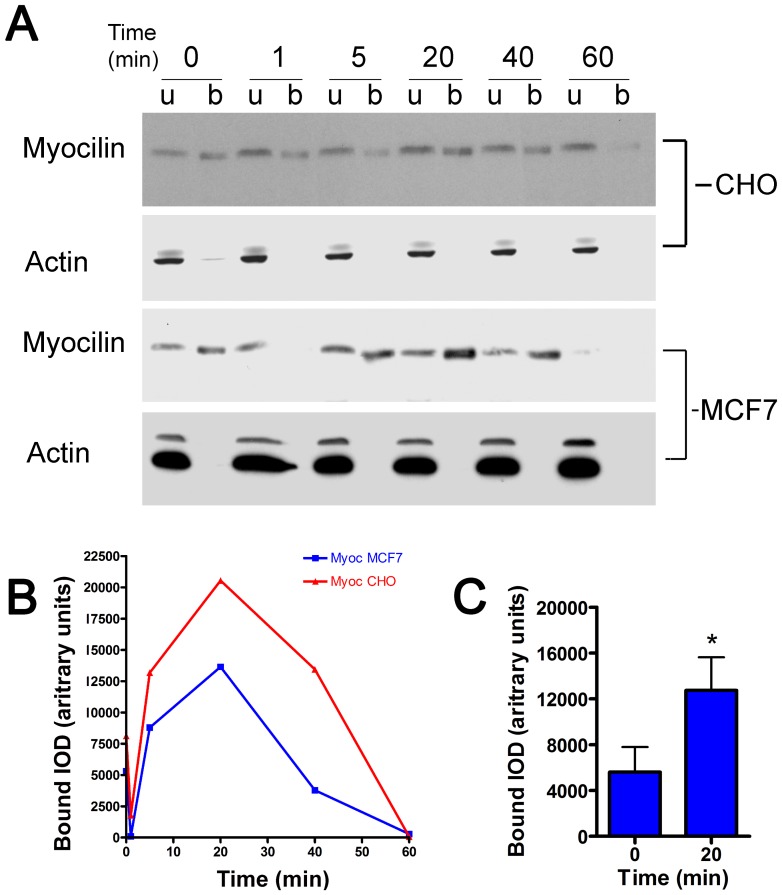
Time-course of ligand-dependent myocilin recruitment to cell-surface membranes containing GPR-143. After labeling cell surface proteins with biotin, CHO and MCF7 cells stably expressing MYOC and GPR143 were stimulated with l-DOPA, then proteins were harvested at the times illustrated. (A). Shown are representative western blots of cell lysates subjected to strepavidin chromatography. Proteins in bound (b) and unbound (u) fractions were probed for myocilin (MYOC) and actin abundance. (B). Specific bands from blots were analyzed using ImageJ to quantify band intensity and kinetic results of bound fraction for all three proteins analyzed for an individual experiment is shown. Results are representative of 4 experiments for each cell type tested. The mean values of the bound MYOC at time zero and at 20 min are compared for the experiments in aggregate, and found to be significantly different (* p<0.05).

To more closely examine myocilin involvement in GPCR internalization, two heterologous expression systems were utilized. CHO and MCF7 cells were transfected to express both myocilin and GPR143. Mimicking behavior of different cell types *in vivo*, MCF7 cells released recombinant myocilin extracellularly, while CHO cells did not (not shown). Cell surfaces of transfected cells were biotinylated, then cells were stimulated with 1.0 µM l-DOPA and proteins were harvested over time. Unbound and bound protein fractions were analyzed by western blot, probing for myocilin, arrestin-3 and actin. Results show a recruitment of myocilin to the biotinylated membrane protein fraction, beginning at 5 minutes, peaking at 20 minutes and decreasing thereafter (Figure 4A). We also noted that in each experiment the initial level of myocilin in the bound fraction was greater at time 0 than at the next minute, most likely due to mechanical perturbation of the membrane during the rapid media change. This action is known to cause a rapid transient [Ca^++^]_I_ which is normalized by a minute.[41] Overall, the amount bound at 1 minute was approximately 20% lower than that at time zero, but this difference was not statistically significant. Densitometric analysis of western blots shows kinetics for protein abundance in bound fraction over time (Figure 4B). Regardless of the cell type studied, results were similar. When the amount of myocilin present in the bound fraction at time zero and 20 minutes after stimulation with l-DOPA were compared from 8 individual experiments, the proportion of myocilin associated with biotinylated membrane proteins significantly increased by 197.4% (p<0.05, Figure 4C). We also investigated arrestin 3 recruitment by western blot and observed a similar transient recruitment to the biotinylated protein fraction (not shown).

### Myocilin mutations that cause glaucoma exhibit different kinetics than WT myocilin

**Figure 5:**
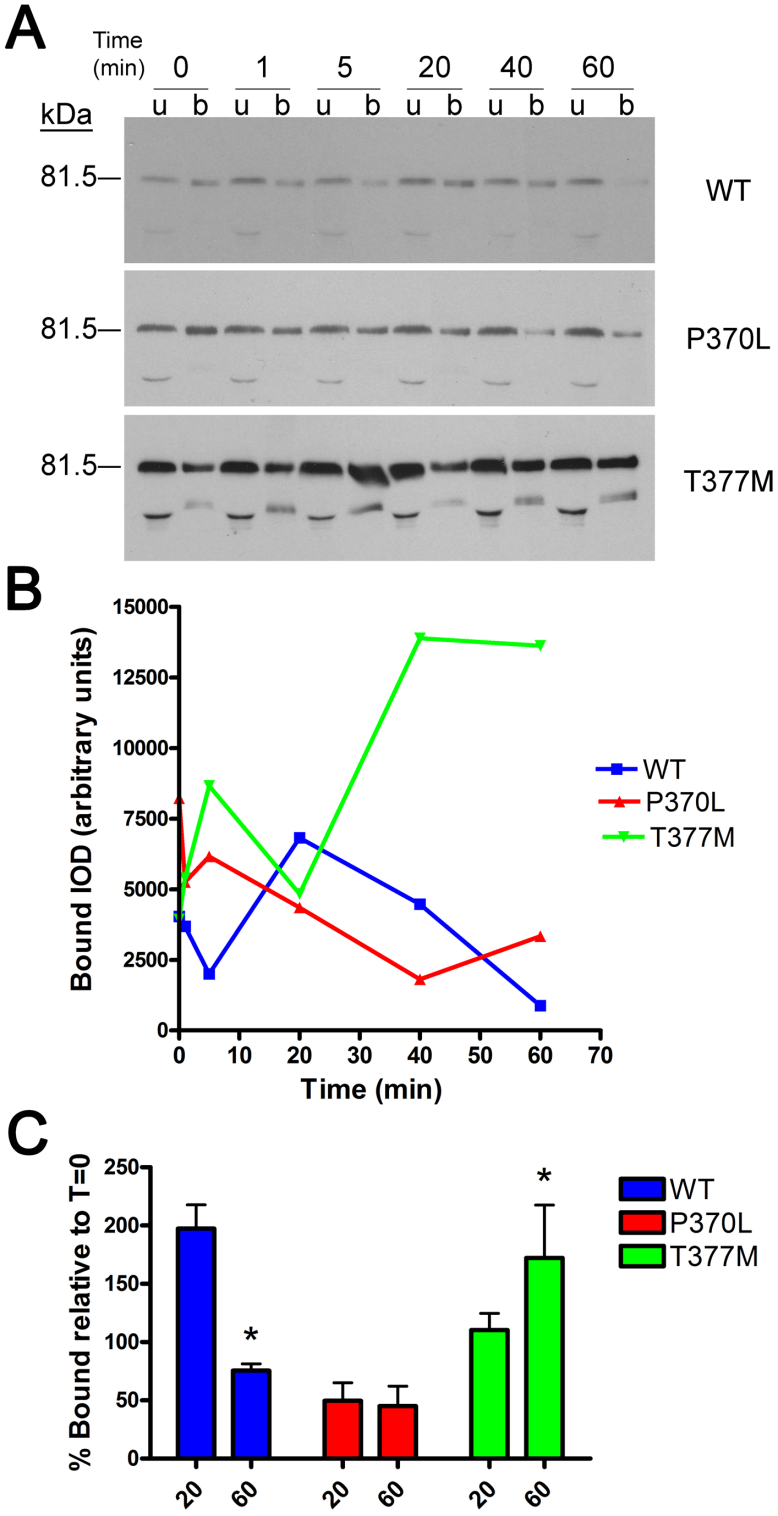
Glaucoma-associated mutations alter ligand-stimulated recruitment of MYOC to cell surface membranes containing GPR-143. Cell surface proteins of CHO and MCF7 cells stably expressing GPR-143 and MYOC (WT, P370L or T377M) were first biotinylated then cells were treated with L-DOPA for the times indicated. (A) Biotinylated proteins were captured (bound, b) using streptavidin chromatography and distribution of myocilin (MYOC), and actin compared to unbound (u) using western blot analyses. Panel B compares densitometric analysis of band intensities of myocilin protein isoforms in bound fraction over time. Shown is a representative experiment, repeated a total of eight times (4 times each in CHO and MCF7 cells). In panel C the mean (± SD) amount of MYOC in the bound fraction are compared at 2 time points, 20 and 60 minutes from 8 experiments (* p<0.05).

Since mutations in myocilin cause glaucoma, two disease-causing point mutations were tested for their impact on l-DOPA-stimulated recruitment of myocilin to the “bound” protein fraction. CHO or MCF7 cells were transfected to express GPR143 plus wild-type (WT) myocilin or mutant myocilin (P370L or T377M). Results show that the two mutants differed in their behavior from WT MYOC and from each other (Figure 5). For example, western blot analysis of P370L demonstrated no significant recruitment to the bound protein fraction after L-DOPA treatment. Interestingly, P370L presence in the bound fraction actually decreased over time. In contrast, the T377M myocilin isoform content immediately increased after stimulation of cells with L-DOPA, and continued to increase until the final 60-minute time point. Densitometric analyses of western blots illustrate the diverse kinetics observed between the three different myocilin isoforms (Figure 5B). The mean bound protein abundance at 20 and 60 minutes after L-DOPA treatment relative to the time zero was compared between isoforms (Figure 5C). Data show that the amount of bound P370L isoform was less than control at both 20 and 60 minutes. On the contrary, the amount of T377M isoform was slightly greater 20 minutes after treatment and significantly greater after 60 minutes (172.9%, p<0.05).

### Myocilin associates with the cytoplasmic domain of GPR143

**Figure 6:**
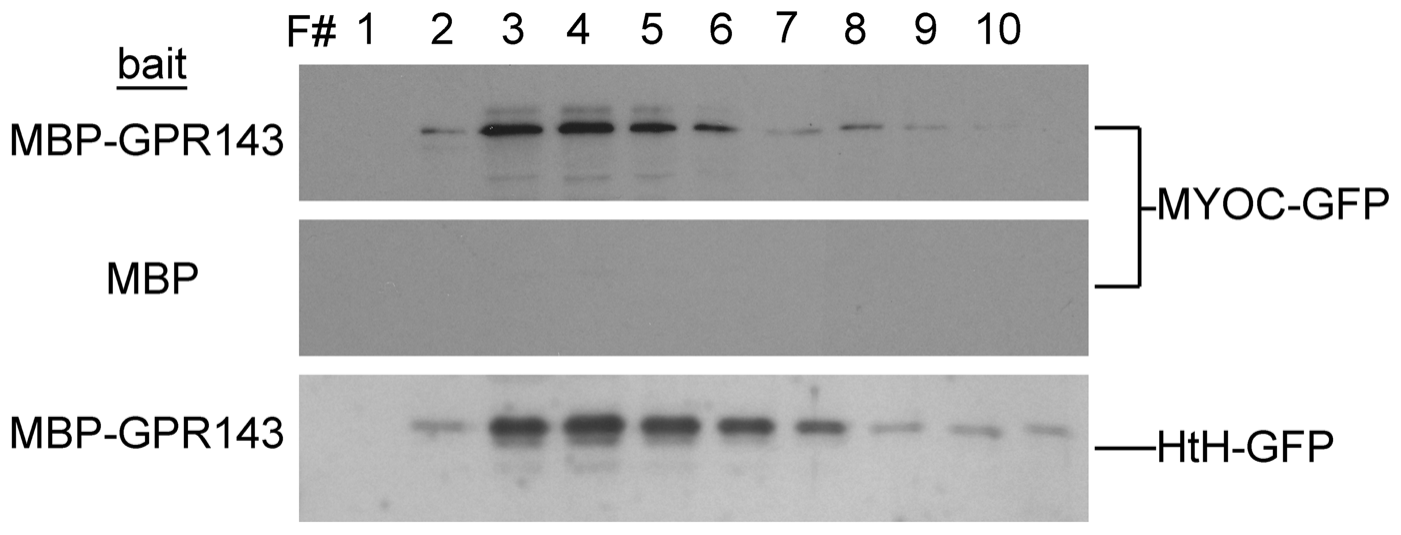
Interaction between myocilin and the cytoplasmic domain of GPR143. Recombinant GPR143-maltose binding protein (MBP) fusion protein or MBP alone were bound to immobilized amylose and lysates from COS cells expressing full-length myocilin tagged with GFP (MYOC-GFP) or the HTH domain (N-terminal 58 amino acids, HTH-GFP) of myocilin were passed over the columns. Columns were washed and bound proteins were eluted by competition with excess maltose. Shown are western blot analysis of elution fractions from columns containing immobilized GPR143:MBP or MBP. Results are representative of 3 experiments with each construct.

To test the hypothesis that myocilin enters the membrane compartment bound to a GPCR, a construct that contained the cytoplasmic domain of GPR143 fused to maltose binding protein (GPR143:MBP) was generated. The construct was expressed in bacteria, immobilized on amylose resin and then lysates from cells expressing WT myocilin fused to GFP (MYOC:GFP) were passed over column containing GPR143-MBP. After washes, interacting proteins were eluted with excess maltose, fractions collected and analyzed for myocilin content by western blot. Results illustrate that myocilin specifically associates with GPR143:MBP, but not to immobilized MBP (Figure 6) nor did GFP bind to immobilized MBP (not shown). To determine the folding domain of myocilin [12] that mediates the association with the GPR143:MBP, the amino helix-turn-helix domain which encompasses the first 58 residues of myocilin fused to GFP (HTH-GFP) was tested. Similar to full-length myocilin, HTH-GFP associated with GPR143:MBP (Figure 6), but not MBP (not shown).

### Characterization of the properties of myocilin bound to GPR143

**Figure 7:**
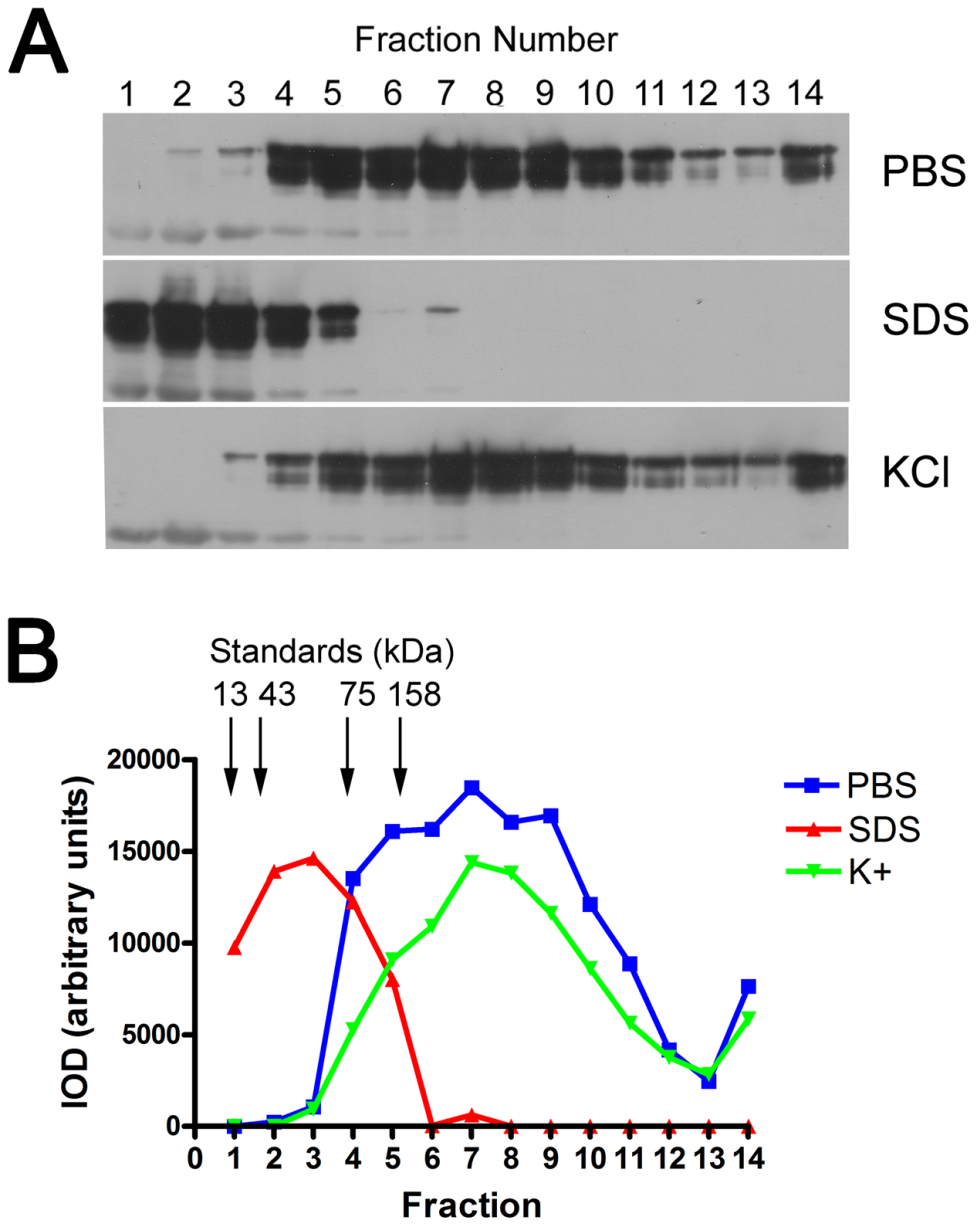
Myocilin is part of a large complex of proteins bound to the cytoplasmic domain of GPR143. Elution fractions 3-6, containing WT MYOC from GPR143:MBP column were pooled and maintained in PBS prior to sedimentation, or adjusted to 0.1% SDS or 500 mM KCl to determine the stability of the protein complex. Samples were then subjected to glycerol gradient sedimentation, SDS-PAGE and analyzed by western blot (panel A). Densitometric analysis of the relative MYOC band intensities in the various fractions is displayed in panel B. Parallel gradients were run with proteins of known molecular weights, which were visualized by silver stain (not shown). The peaks of elution profiles for the standards are indicated with arrows and corresponding molecular weight. Results shown are from a single experiment that is representative of 3 experiments in total.

In the previous experiment we have shown interaction between myocilin and the cytoplasmic domain of GPR143, suggesting that myocilin could arrive at the membrane compartment bound to a GPCR. To determine the stability and properties of the myocilin:GPR143 interaction, fractions 3-6 from cell lysates containing myocilin-GFP eluted from a GPR143:MBP column were combined, split into three portions and then adjusted to three different conditions: (i) PBS, (ii) 0.1% SDS or (iii) 500 mM KCl, final concentrations. The three solutions were then subjected to glycerol gradient sedimentation. Results in figure 7 show that myocilin associated with the GRP143:MBP as part of a protein complex of proteins (>158 kD), larger than the myocilin dimer (110 kD). Unlike the myocilin complex observed in membrane fractions from human tissues [18], this complex was dissociated by SDS, which resulted in myocilin at the apparent size of a monomer (55 kD). Next, the ionic sensitivity of the complex was examined using high K^+^ and found to be stable in 500 mM KCl. A comparison of elution profiles based upon densitometric analysis of myocilin western blot bands from each of the three conditions is shown in figure 7B.

## Discussion

The present study identifies for the first time the cellular pathway in which myocilin functions. Four complementary lines of evidence are shown to support a role for myocilin in ligand-mediated GPCR internalization: Upon activation by ligand, recombinant myocilin in two different cell lines *in vitro* is recruited to endocytic vesicle membranes containing GPR143 and arrestin-3 or arrestin-2 GFP. In recombinant models, the interaction was detected both biochemically and via confocal imaging of tagged proteins illustrating that the vesicles were endosomes derived from the cell surface. Significantly, two different point mutations in myocilin that cause glaucoma differentially disrupt the character and timing of myocilin-GPCR interactions. Lastly, the cytoplasmic tail of GPR143 and the amino terminal domain of myocilin specifically interact as part of a large protein complex.

As a glaucoma protein, myocilin is not alone in its association with the endocytic pathway. Recent data suggest that three other gene products that associate with glaucoma function in the endocytic pathway. WDR36 (PMID:21940795) [42], optineurin (also known as FIP-2 [43]–[45], PMID:16091361) [46], [47] and tank binding protein kinase-1 (PMID:18307994) [48]–[52] have all been recently implicated in cellular processes associated with the endocytic pathway. Importantly, both WDR36 and optineurin have been shown to interact with and modulate G-protein-coupled receptor signaling. Optineurin participates in desensitization of Group I metabotropic glutamate receptors [53], [54], while WDR36 acts as a scaffold holding thromboxane A2 receptors and Gαq signaling complex [55]. Like optineurin and WDR36, here in the present study we show myocilin associates with a Gαq-coupled receptor, GPR143, which increases [Ca^++^]_I_. The slight decrease observed between time zero after a rapid media change and the 1 minute time point may also be [Ca^++^]_I_ related, and may suggest that myocilin association at the membrane may be Ca^++^ -dependent.

GPR143 was chosen as a candidate protein to test our hypothesis about myocilin and receptor internalization for two main reasons: First, we were able to utilize our previous experience and custom made reagents working with GPR143 [39]. Second, GPR143 is exquisitely sensitive to the presence of its endogenous ligands, l-DOPA or tyrosine, resulting in rapid internalization [39]. In fact, early studies mistakenly reported that GPR143 was the only intracellular GPCR [56]. Recently, however, we demonstrated that tyrosine in cell culture media was responsible for its apparent intracellular distribution [39]. Once tyrosine is removed from culture media, GPR143 appears on the cell surface like other GPCRs. Retinal pigment epithelial cells (RPE) express both GPR143 and myocilin making that pairing relevant as they are present in the same cells *in vivo.* However, results using this model suggest promiscuity by myocilin since myocilin is widely expressed but GPR143 expression is limited to pigmented cells. It is likely that myocilin interacts with other types of surface receptors where persistent activation triggers endocytosis. Given the universal nature of endocytosis and the very limited phenotype associated with myocilin glaucoma, some level of cell-type or tissue specificity is likely. Further studies are required to determine the scope of receptors with which myocilin interacts, in particular the specific receptor(s) in ocular cells involved in myocilin glaucoma.

To examine the timing and kinetics of myocilin association with biotinylated cell surface proteins we used recombinant proteins in cultured cells. We specifically chose two different cell types, one that releases myocilin into the extracellular space, MCF7, and another that does not, CHO. Interestingly, results from all assays and paradigms examining endocytosis using both cell types were quite similar which suggests that, whether the cell or tissue releases MYOC on exosomes, its role in receptor-mediated endocytosis is similar and independent from whether the cells release the protein. Results from the 2 cell types were pooled together for statistical analysis since, while individual experiments varied based on the level of protein expression, there was no significant difference in response between the cell types when comparing the 4 experiments between MCF7 with the 4 with CHO cells. Analysis of wild type myocilin co-localization with arrestin 2 indicate myocilin is retained with the endosomes after arrestin 2 is released, suggesting early sorting steps occur and myocilin is retained with the GPCR. Arrestin proteins are very specific for GPCR endocytosis, and only interact with activated GPCRs during receptor-mediated endocytosis for a short time after internalization. Arrestin is lost from the vesicles prior to myocilin suggesting myocilin traffics with the receptor further into the endosomal sorting process than arrestin. Functionally, this may indicate that myocilin is recruited to and enters the vesicular pathway during receptor-mediated endocytosis like arrestin- a known scaffolding protein, but our data also indicate that myocilin traffics differently than both arrestin and GPR143 on its way to the MVB and subsequent release on exosomes.

One weakness of our biotinylation approach is that for us to observe myocilin in the bound fraction it must still be associated with a cell surface biotinylated protein, and this changes as endosomal sorting continues. Once myocilin is released from the membrane protein, we loose our ability to track it further along the endosomal pathway. Further experiments will be necessary to follow myocilin into the MVB, determine when and how it becomes associated with exosomes and is released from the cell.

Myocilin binding to biotinylated plasma membrane proteins shows that myocilin interaction peaked at 20 minutes, but was dissociating by 40 minutes, indicating myocilin is likely retained on the endosome with the GPCR during early sorting, as suggested by the microscopy data at 40 minutes showing myocilin and GPR143 in separate vesicles. We also noted that specifically in MCF7 cells there appeared less total myocilin at sixty minutes, but this was not statistically significant over the 4 experiments. With respect to myocilin mutants, we chose to study a severe early onset mutation, P370L [57], and compare that to an adult onset mutation, T377M [9]. We found that the P370L mutant was not recruited to GPR143 in response to L-DOPA. However, P370L was observed in the bound fraction even prior to ligand expoure, perhaps illustrating it is not releasing from an autocrine interaction. In contrast, the conservative T377M mutant associated with biotinylated plasma membrane proteins, but the kinetics did not match the WT. Instead, the T377M isoform more efficiently bound to biotinylated plasma membrane proteins, demonstrated recruitment after ligand stimulation but failed to dissociate from the biotinylated proteins like WT. While both WT and T377M were found associated with the biotinylated plasma membrane proteins and released into the culture media from MCF7 cells, the P370L isoform was not, and showed little association with plasma membrane proteins. Results suggest that myocilin entry into the exosome pathway begins at receptor endocytosis, and failure to enter the endosomal pathway at that point results in cellular retention of the protein. Furthermore, sorting of endosomal proteins and exosome biogenesis appear to be separate events and likely cell-type and context dependent.

Our experiments are limited by the fact that we used one cell type that endogenously produces myocilin (MCF7) and another that for which endogenous myocilin expression in unknown (CHO). In our experiments we used recombinant methods to over express arrestin-2, GPR143, and myocilin which could lead to spurious associations. In the course of these experiments we used both GPR143 and myocilin as GFP fusions, but we also localized both proteins by immunofluorescence as nonfusion proteins. We observed the same localization pattern either way indicating that the GFP fusion was not creating the localization and protein interactions we observed. We also used recombinant protein capture studies to illustrate that the amino terminus of myocilin binds to the cytoplasmic tail of GPR143, indicating that myocilin binds to a GPCR. In totality, our data strongly indicate that myocilin associates with the cytoplasmic domain of a GPCR in a ligand dependent manner.

Myocilin has three domains, with a short 58 amino acid HTH amino terminal domain, a central coiled-coil domain, and a globular carboxyl terminus domain with homology to the olfactomedin protein family. In chromatography experiments, the purified carboxyl tail of GPR143 captured both the HTH domain alone, as part of a large complex, and a protein complex containing full-length myocilin. The complex was fairly stable, surviving protein purification and glycerol gradient sedimentation, and was not dissociated in high potassium. When comparing the high salt and normal PBS we did not observe ‘free’ myocilin similar to previous results from human tissue showing that membrane-associated myocilin was ***only*** present as part of a complex [18]. However, unlike previous results with endogenous myocilin from a total cell membrane preparation, the complex observed in this study was disrupted by SDS, yielding a myocilin monomer. The myocilin-GPR143 complex identified here is larger than the highest molecular weight marker used (158 kD) but smaller than the ∼405 kD complex observed in human tissue samples, and differs in its SDS sensitivity. Hence, we either assembled a protein complex *de novo* on the purified GPCR tail, or captured a pre-assembled myocilin complex from the cells that may be a precursor to the larger membrane associated SDS-resistant complex. Further studies are required to resolve these issues.

Results from the present study showing a role for myocilin in the endocytic pathway provide an explanation for some incongruent results in the literature. The central issue is whether myocilin enters the endoplasmic reticulum (ER) and is secreted in a traditional fashion, or is a cytosolic protein that associates with the vesicular compartment. Due to the interconnectivity of membrane compartments via vesicular traffic, dominant negative effects by myocilin in the endocytic pathway would likely impact function of vesicle producing organelles, including the ER; particularly in models where mutant myocilin is dramatically overexpressed [58]–[62]. In fact, recent studies demonstrate the interdependent relationship between early endosomes and autophagy/ER stress pathways are thought to mediate mutant myocilin pathology [63], [64]. Reports of mutant myocilin causing ER stress in some cells and animal models is difficult to interpret because of dramatic overexpression. For example, transgenic mice expressing mutant mouse myocilin at physiological levels do not display elevated IOP and ER stress [65], although transgenic mice overexpressing (>10 fold) mutant human myocilin have elevated IOP and show signs of ER stress [66]. It is unclear whether overexpression of mutant myocilin or human/mouse versions of the protein are responsible for the ER stress and pathology. Regardless, if expression of mutant myocilin causes ER stress and cell death in humans, then it is perplexing why pathology localizes to the eye, and not heart and skeletal muscle which also express comparable levels of myocilin [67].

As such, myocilin is a widely expressed protein that probably has a similar function in most cell types, but mutations lead to a very limited phenotype, ocular hypertensive glaucoma. To understand myocilin pathology, our results here suggest two fundamental directions to pursue: One possibility is that myocilin mutations alter receptor endocytosis, which impacts receptor down regulation and produces defective intracellular signaling. Receptor specificity may then account for selective cellular disruption. The second possibility is that mutant forms of myocilin selectively alter exosome formation and/or release from cells of the aqueous humor outflow pathway. Maybe mutant forms of myocilin alter extracellular communication pathways that are dependent upon selective expression of receptor internalization in trabecular meshwork cells. A better understanding of the level at which myocilin disrupts normal cell physiology will provide a novel therapeutic opportunity for the treatment of glaucoma.
